# Testing technology for tensile properties of metal materials based on deep learning model

**DOI:** 10.3389/fnbot.2022.1000646

**Published:** 2022-09-15

**Authors:** Xuewen Chen, Weizhong Fan

**Affiliations:** ^1^Guangdong Engineering Polytechnic College, Guangzhou, China; ^2^Huajin New Materials Research Institute (Guangzhou) Co., Ltd., Guangzhou, China; ^3^Guangdong Hongbang Metal Aluminum Co., Ltd., Guangzhou, China

**Keywords:** neural Turing machine, metallic material, tensile experiment, inspection technique, hard sigmoid

## Abstract

The properties of metallic materials have been extensively studied, and nowadays the tensile properties testing techniques of metallic materials still have not found a suitable research method. In this paper, the neural Turing machine model is first applied to explore the tensile properties of metallic materials and its usability is demonstrated. Then the neural Turing machine model was improved. The model is then improved so that the required results can be obtained faster and more explicitly. Based on the improved Neural Turing Machine model in the exploration of tensile properties of metal materials, it was found that both H-NTM and AH-NTM have less training time than NTM. A-NTM takes more training time than AH-NTM. The improvement reduces the training time of the model. In replication, addition, and multiplication, the training time is reduced by 6.0, 8.8, and 7.3%, respectively. When the indentation interval is 0.5–0.7 mm, the error of the initial indentation data is large. The error of the tensile properties of the material obtained after removing the data at this time is significantly reduced. When the indentation interval is 0.8–1.5 mm, the stress is closer to the real value of tensile test yield strength 219.9 Mpa and tensile test tensile strength 258.8 Mpa. this paper will improve the neural Turing machine model in the exploration of metal material tensile properties testing technology has some application value.

## Introduction

As a material, metallic materials are widely used in human production, life and social development (Stock et al., 2018). Metallic materials have many properties, such as high elasticity, high toughness, and high hardness (Kumar et al., 2020). In the metal industry, metallic materials are usually divided into pure metals and alloys (Suryanarayana, 2019). The physical properties of metal materials are usually tested when making the corresponding materials and equipment. The physical testing of metals is carried out according to industry standards and using scientific methods. Therefore, it is necessary to strengthen the research on the physical properties testing technology of metals and to improve the corresponding technical measures (Xu et al., 2021).

The effects on the properties of metallic materials are usually reflected within the metallic material. When people process metal materials, the metal material is easily affected by the tensile speed. Generally metal inclusions, metal crystals and other impurities are present inside the metallic material. These magazines lead to problems such as crystal misalignment and poor bonding within the metallic material (Ford et al., 2019). Metallic materials generally have a relatively consistent overall performance, but the tensile properties of metallic materials will be affected when external elastic deformation or plastic deformation occurs during processing (Khalid et al., 2022). According to Regan et al. (2020), who studied plastic materials, it was found that plastic deformation of materials can be accomplished by stretching. The external processing can cause the relative sliding of the metal material beyond the slip threshold, and this phenomenon will cause the crystalline and crystallographic motion of the metal crystal. This process will have a velocity of motion. When the metal material is stretched, the strength will also increase when the stretching temperature increases, and there will be a time lag in the stretching process. At a slow rate of stretching, the technical material can withstand a tensile force of 200 kN.

When the stretching speed is increased, applying 200 kN tension to the metal material will cause dislocation intensive reduction of the material tensile properties and fracture of the metal material. Yuan and Fan (2019) found that a reasonable choice of speed and pressure is required when stretching metal materials. When the metal is stretched, this operation needs to ensure that the metal crystal slip is produced and the tensile properties of the material are taken into account. And to avoid the fracture of the metal material during the operation. The properties of metallic materials have been widely studied, but for their tensile properties testing techniques, they are currently difficult to find a suitable method for researchers to explore simple, accurate and fast testing techniques for the tensile properties of metallic materials. In today's era of exponential growth of data, the value laws behind the data are often buried under the vast amount of information. How to uncover the potential value through the surface phenomenon and exploit it has become the focus of current technology research (Bai et al., 2022). Yao and Guan (2018) stated that natural language processing is a popular area of data research. In terms of algorithm implementation, machine learning methods have received wide attention from scholars both at home and abroad. Neural networks have features such as automatic feature extraction and strong description ability (Ning et al., 2022). Among many machine learning methods, neural networks have become a dark horse in the machine learning community. Neural networks have made breakthroughs in many research areas. In the research of Neural Turing Machine (NTM), a Neural Turing Machine (NTM) is a kind of neural network with Turing-complete properties. It has the ability to fit functions and can theoretically implement any function. According to Gangal et al. (2021), it was found that the most important difference between NTM and physical Turing machines is that a Neural Turing Machine is an algorithm that can pass gradients backwards. The physical concept of a Turing machine uses the 0 or 1 representation of data in a computer to compute all logical functions (Malekmohamadi Faradonbe et al., 2020). The same feature as all algorithms is that neural Turing machines, like all neural network algorithms, use mainly real numbers (Boce et al., 2022). Neural Turing machines use activation functions with smoother function images to make the neural network properties appear continuously non-linear. Such non-linear neural networks composed of real numbers are easier to train (Huang et al., 2020). Neural Turing machines combine physical Turing machine ideas and smooth activation functions to perform the operations associated in physical Turing machines. Another difference from physical Turing machines is that physical Turing machines read the instructions to be executed continuously in one direction in a sequential manner (Mühlhoff, 2020). In contrast, during the addressing of a neural Turing machine, the neural Turing machine can computationally generate a displacement that shifts the center of gravity of the current attention to the left or right, rather than simply to one direction (Faradonbeh and Safi-Esfahani, 2019). The focus of NTM is on the management of external memory. NTM extends the functionality of standard controllers by reading and writing external memory as a result of addressing. Thus, they can make the NTM implement the memory management function. According to Sharma et al. (2020), it was found that the addressing mechanism of NTM also makes the controller in NTM to generate certain attention. Thus, NTM can improve the model's ability to process sequences. Deep Reinforcement Leaning (DRL) is used to solve the problem of too many states in reinforcement learning (Wang et al., 2022). Deep reinforcement learning methods construct a function with parameters to fit the value assessment of state actions (Quan et al., 2020). Deep reinforcement learning obtains action chains with corresponding values by trying different strategies, which in turn can tune the parameters of the value function. Thus, they can make the prediction of the value function converge to the actual value (Bai et al., 2021). It has also become a trend to add deep learning to NTM as the optimal strategy can be obtained through the value function (Wang et al., 2021). In the study by Gross et al. (2021), this study used the NTM mechanism to improve the network model structure. A data copy experiment and a data repetitive copy experiment were designed in the study. The effectiveness of the attention mechanism generated by NTM was verified from the experimental results. The metal material tensile property testing technique has been widely explored, so combining neural network applied to metal material tensile property testing technique is rarely studied and the applicability study under this combined neural Turing model is almost absent.

In summary, this problem is explored for the tensile properties testing technique of metal materials. In this study, an improved neural Turing machine model is proposed. The model uses the Hard sigmoid function instead of the sigmoid activation function in NTM. This approach makes the model computationally simple and easy to optimize. This approach ensures that the core structure of the NTM remains unchanged, while reducing the computational effort of the model and speeding up the model training. In this paper, the improved neural Turing machine model is applied to the problem of exploring the tensile properties testing technology for metal materials. In the study, it is found that the improved neural Turing machine can reduce the training time of the model. When the indentation interval of metal material is 0.5–0.7 mm, the error of the tensile property results obtained after removing the initial indentation data is significantly reduced and is closer to the real value. When the indentation interval is 0.8–1.5 mm, the accuracy of fitting the results using the default range is higher.

## System model

### Introduction to the neural turing machine model and formulas

#### Introduction of neural turing machine

A neural Turing machine is a neural network architecture with the addition of an external storage matrix. The external storage matrix enhances the neural network's ability to remember long input sequences, forming an attention mechanism similar to the Seq2Seq model. This external memory-based architecture is consistent with computer Turing machines. Only in contrast to computer Turing machines, an end-to-end microscopic neural network model of NTM can be trained using gradient descent method for network modeling.

The main components of the NTM are the controller, the read/write side, and an external memory (Urien, 2019). The controller in the NTM is equivalent to the CPU in a computer, and the external memory is equivalent to the memory of a computer. The read/write side is equivalent to the IO device of the computer. The controller modifies and reads the memory blocks through the read/write side. During the operation of the computer, the CPU addresses the data according to the control signals from the controller, and the CPU determines where in the memory to read and write the data information. Unlike actual machines, there is no concept of bootability for computer operations on memory. In NTM, all read and write operations to the memory block matrix are derivable (Vishwakarma and Lee, 2018).

The output of the NTM controller controls the entire workflow of the NTM. The implementation of the controller is a neural network. This means that it can be a recurrent neural network. It can also be a fully connected or convolutional network. It is the neural network controller that interacts with the entire system input and output. The read and write sides of the NTM calculate the weights of each vector in the external memory matrix for the current state based on the control signals from the controller. The values of the memory matrix in the NTM are affected by all the inputs up to the current moment. the memory matrix in the NTM is a real matrix. the memory matrix in the NTM is the object of direct operations by the read and write side. The process of reading and writing against the memory matrix in the sequence model is represented as an attention mechanism.

#### Formulation of the neural turing machine

*M*^(*t*)^ denotes the memory matrix of size N × E at moment *t*, where *N* denotes the number of memory cells and E denotes the size of each memory cell. *w*^(*t*)^ denotes the weight vector output through the read head at the moment of *t*. *w*^(*t*)^ whose the *i*-th dimensional element $w_{i}^{\left(t\right)}$ represents the weight occupied by the *i*-th memory cell and satisfies the following constraint.


(1) $$\begin{array}{l} \overset{N}{\underset{i=1}{\sum }}w_{i}^{\left(t\right)}=1,0\leq w_{i}^{\left(t\right)}\leq 1,i=1,2,\ldots N \end{array}$$

Then the reading vector *r*^(*t*)^ at moment t is calculated according to Equation (2).


(2) $$\begin{array}{l} r^{\left(t\right)}=w^{\left(t\right)}M^{\left(t\right)} \end{array}$$

At the moment of *t*, the write head outputs the weight vector *w*^(*t*)^, the E dimensional elimination vector *e*^(*t*)^ and the E dimensional *a*^(*t*)^. *e*^(*t*)^ each element belongs to the interval (0, 1). Then the value of the memory matrix can be calculated according to Equations (3)–(5) as follows:


(3) $$\begin{array}{lll} e^{\left(t\right)} & = & \sigma \left(W^{e}h^{\left(t\right)}+b^{e}\right) \end{array}$$

(4) $$\begin{array}{lll} a^{\left(t\right)} & = & W^{a}h^{\left(t\right)}+b^{a} \end{array}$$

(5) $$\begin{array}{lll} M^{\left(t\right)} & = & M^{\left(t-1\right)}|1-w^{\left(t\right)}{\left(e^{\left(t\right)}\right)}^{T}|+w^{\left(t\right)}{\left(a^{\left(t\right)}\right)}^{T} \end{array}$$

where, 1 in Equation (5) denotes an all-1 matrix of size N × E, denotes the output of the controller at the moment of *t*, *W*^*e*^, *b*^*e*^, *W*^*a*^, and *b*^*a*^ are the weights and biases corresponding to the elimination vector and the additive vector, respectively. From Equation (5), it can be seen that each element of the memory matrix is reset to 0 when each element of *e*^(*t*)^ and *w*^(*t*)^is equal to 1, and then a new memory vector is written.

When each element of *e*^(*t*)^ and *w*^(*t*)^ is equal to 0, each element of the memory matrix remains unchanged.

The addressing mechanism based on location addressing is introduced into NTM. In this paper, the addressing mechanism of NTM, that is, the weight vector occupied by the ith memory unit, is summarized as the following four formulas:


(6) $$\begin{array}{lll} C_{i}^{\left(t\right)} & = & \frac{\text{exp}\left(\beta^{\left(t\right)}k\left(k^{\left(t\right)},M_{i}^{\left(t\right)}\right)\right)}{\underset{j}{\sum }\text{exp}\left(\beta^{\left(t\right)}k\left(k^{\left(t\right)},M_{i}^{\left(t\right)}\right)\right)} \end{array}$$

(7) $$\begin{array}{lll} G_{i}^{\left(t\right)} & = & g^{\left(t\right)}C_{i}^{\left(t\right)}+\left(1-g^{\left(t\right)}\right)w_{i}^{\left(t-1\right)} \end{array}$$

(8) $$\begin{array}{lll} {\tilde{w}}_{i}^{\left(t\right)} & = & \overset{N-1}{\underset{j=0}{\sum }}G_{j}^{\left(t\right)}s_{i-j}^{\left(t\right)} \end{array}$$

(9) $$\begin{array}{lll} w_{i}^{\left(t\right)} & = & \frac{{\tilde{w}}_{i}^{\left(t\right)y\left(t\right)}}{\underset{j}{\sum }{\tilde{w}}_{j}^{\left(t\right)\gamma \left(t\right)}} \end{array}$$

The *K* function in Equation (6) represents the cosine similarity function.


(10) $$\begin{array}{l} k\left(u,v\right)=\frac{u\cdot v}{\left||u||||v|\right|} \end{array}$$

Equations (6) and (9) involves the five parameters *k*^(*t*)^, β^(*t*)^, *g*^(*t*)^, *s*^(*t*)^ ,γ^(*t*)^ and according to the previous section, have their specific physical meaning. In the structure of NTM, they each correspond to a single layer of neural networks whose inputs are controller outputs *h*^(*t*)^. Where *k*^(*t*)^ corresponds to a linear activation function, β^(*t*)^, *g*^(*t*)^, *s*^(*t*)^, γ^(*t*)^ corresponding to the activation functions 1+ReLU, sigmoid, softmax, and 1+ReLU, respectively. the following equation gives the definition of these five parameters:


(11) $$\begin{array}{lll} k^{\left(t\right)} & = & W^{k}h^{\left(t\right)}+b^{k} \end{array}$$

(12) $$\begin{array}{lll} \beta^{\left(t\right)} & = & 1 + ReLU\left(W^{\beta }h^{\left(t\right)}+b^{\beta }\right) \end{array}$$

(13) $$\begin{array}{lll} g^{\left(t\right)} & = & \mathrm{sigmoid}\left(W^{g}h^{\left(t\right)}+b^{g}\right) \end{array}$$

(14) $$\begin{array}{lll} s^{\left(t\right)} & = & \mathrm{softmax}\left(W^{s}h^{\left(t\right)}+b^{s}\right) \end{array}$$

(15) $$\begin{array}{lll} \gamma^{\left(t\right)} & = & 1+\mathrm{ReLU}\left(W^{\gamma }h^{\left(t\right)}+b^{\gamma }\right) \end{array}$$

shows the output of the time-step t controller, and the W and b appearing in Equation are the weights and biases corresponding to each parameter, respectively.

### Improvement of neural Turing machine

In order to speed up the training of the model, this paper uses hard sigmoid function instead of sigmoid activation function in NTM. Hard sigmoid function has the main properties of sigmoid activation function. Hard sigmoid function also has the characteristics of simple calculation and easy optimization of relu activation function. The hard sigmoid function is used as the activation function, which ensures that the core structure of NTM does not change. It also reduces the calculation of the model.

The relu activation function is defined as follows:


(16) $$\begin{array}{l} g\left(x\right)=\max\left(0,x\right) \end{array}$$

*Relu* is a piecewise linear function. When *x* is a negative number, G (*x*) is equal to zero; When *x* is >0, G (*x*) is equal to *X*.

*Sigmoid* activation function. The activation function related to this article is sigmoid function. It is defined as follows:


(17) $$\begin{array}{l} \sigma \left(x\right)=\frac{1}{1+e^{-x}} \end{array}$$

Its characteristics are: the value range of the function is an interval (0, 1), the function is derivable, and the exp function and division must be calculated for both the function value and the derivative value. Compared with linear function, sigmoid activation function has a huge amount of computation and saturation at both ends of the function.

According to literature (Mao, 2020), this paper notes that Kaiser et al. introduced the truncation mechanism in N-GPUs. Gate truncation mechanism used by Kaiser means that in the gate mechanism of N-GPUs, the sigmoid function is replaced by the function defined in Equation (18).


(18) $$\begin{array}{l} \sigma^{\prime }\left(x\right)=\max\left(0,\min\left(1,1.2\sigma \left(x\right)-0.1\right)\right) \end{array}$$

Hard sigmoid function is a piecewise linear approximation function of sigmoid function. Its definition is shown in Equation (19). According to the introduction of literature (Darabi et al., 2018), this definition comes from courbariaux.


(19) $$\begin{array}{l} \sigma^{\prime }\left(x\right)=\max\left(0,\min\left(1,\left(x+1\right)/2\right)\right) \end{array}$$

### Introduction and formula of tensile properties of metal materials

Tensile properties are one of the important mechanical properties of metallic materials, the yield strength, tensile strength, elongation and sectional shrinkage of metals can be measured by tensile tests and other performance indicators. The relationship between elongation and section shrinkage during the uniform deformation phase has been derived in previous studies under the assumption of constant volume; after necking, the “true stress-strain” curve is also plotted under incorrect strain values.

The stress-strain relationship of the hardening section of power hardening and line hardening metal materials is shown in Equations (20) and (21), respectively.


(20) $$\begin{array}{l} \sigma_{R}=K\varepsilon_{R}^{n} \end{array}$$

*K* is the strength coefficient of power hardening material, which is fitted by the least square method.


(21) $$\begin{array}{l} \sigma_{R}=E_{2}\varepsilon +b \end{array}$$

*E*_2_ is the tangent modulus of linear hardening material, and *b* is the material constant. For metallic materials satisfying the linear elastic power hardening model, the yield strength is the intersection of the elastic deformation line and the plastic deformation curve. Generally, it is determined by the offset of 0.2% of the elastic segment. The tensile strength is calculated by the concept of tensile instability:


(22) $$\begin{array}{lll} \sigma_{y} & = & E\left(\varepsilon_{y}-0.002\right)=K\varepsilon_{y}^{n} \end{array}$$

(23) $$\begin{array}{lll} \sigma_{u} & = & k{\left(\frac{n}{e}\right)}^{n} \end{array}$$

In the formula, the elastic modulus *E* has been calculated by indentation contact stiffness, contact projection circle area and other indentation parameters before calculation. *E* is the base of natural logarithm. For metal materials that meet the line hardening model, the yield strength can be obtained by Meyer's law. The tensile strength is obtained according to the concepts of volume incompressibility and instability during tensile deformation (Ye et al., 2020).


(24) $$\begin{array}{lll} \sigma_{y} & = & \beta_{m}A \end{array}$$

(25) $$\begin{array}{lll} \sigma_{u} & = & \frac{E_{2}}{\text{exp}\left[\left(E_{2}-b\right)/E_{2}\right]} \end{array}$$

*A* is the Meyer index and the material yield parameter, respectively, which is obtained by non-linear regression of the Meyer equation. *m* is the material constant, which is related to the type of metal material. For carbon steel and austenitic stainless steel, this value is usually taken as 0.2285 and 0.1910.

## Analysis and discussion

### Analysis and discussion of neural turing machine

**Figure 1:**
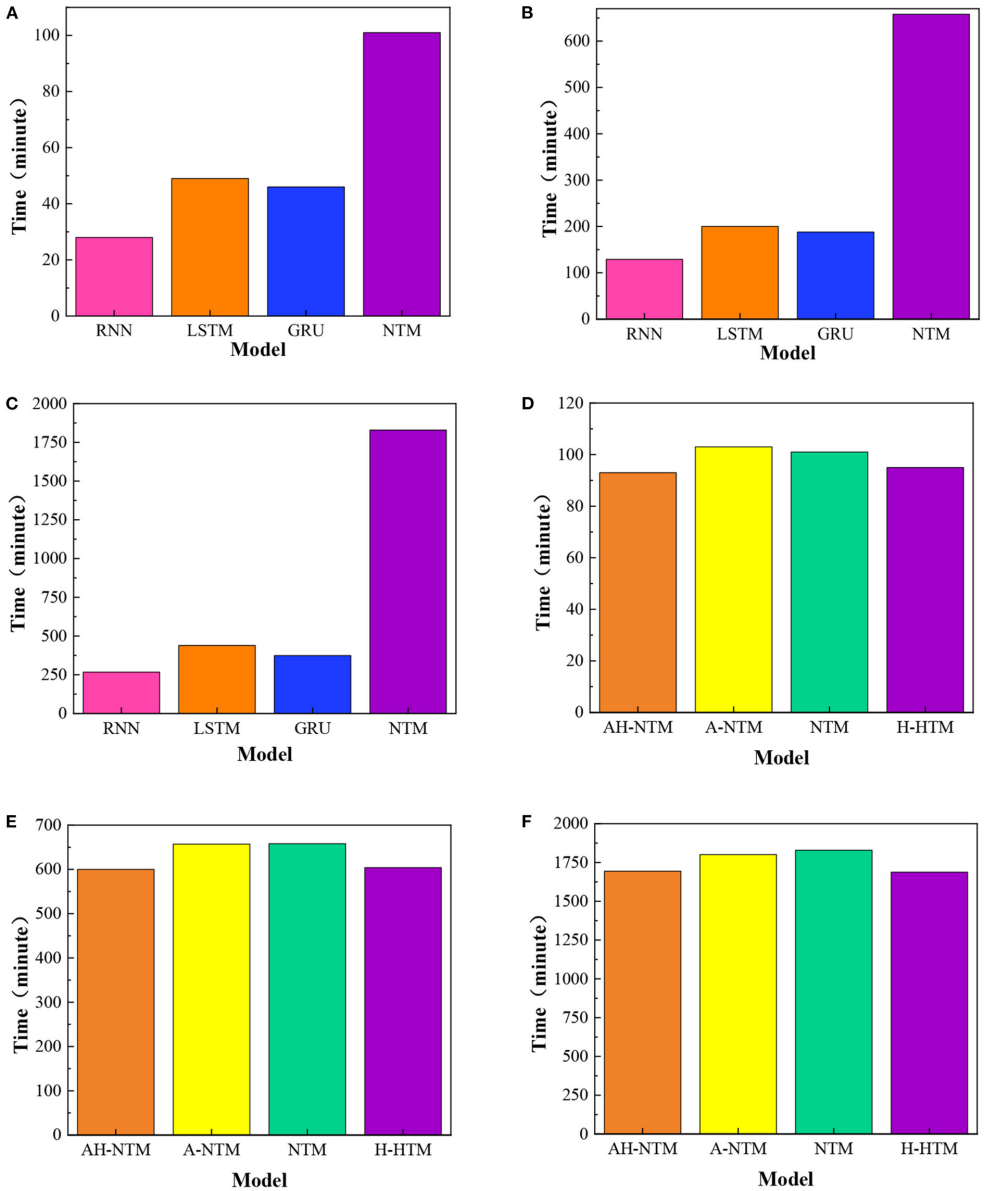
Comparison of training time of different models, **(A)** replication; **(B)** addition; **(C)** multiplication; **(D)** replication; **(E)** addition; **(F)** multiplication.

The experiments introduced in this section compare the performance of different models in algorithm learning tasks, including RNN, LSTM, GRU, and NTM. The experimental tasks include replication, addition and multiplication. Each task trains a model independently. In the copy task, the model trained 50,000 Batches; In the addition task, the model trained 150,000 Batches; In the multiplication task, the model trained 300,000 batches. The performance differences of different models are compared in Figure 1.

The training time of different models is compared in Figures 1A–C. In Figures 1A–C, from top to bottom are copy, addition and multiplication, respectively. The models include RNN, LSTM, GRU, and NTM. The unit of time is minutes. Pink represents RNN, orange represents LSTM, blue represents GRU, and purple represents NTM. It can be seen from the figure that training NTM takes the longest time and training RNN takes the least time. The training time increases in the order of RNN, GRO, LSTM and NTM. In the replication task, it takes 28 min to train RNN, 46 min to train Gru, 49 min to train LSTM, and 101 min to train NTM; In addition to this task, it takes 267 min to train RNN, 188 min to train Gru, 200 min to train LSTM, and 658 min to train NTM; In multiplication tasks, it takes 140 min to train RNN, 374 min to train GRU, 439 min to train LSTM and 1,829 min to train NTM. Although the accuracy of NTM on the test set is higher than GRU, LSTM and NTM, the performance of the model is still relatively poor.

NTM has a long training time. In the replication task, the time required to train NTM is between 2 and 5 times that of other models. In addition to task, the time needed to train NTM is between 3 and 6 times that of other models. In the multiplication task, the time required to train NTM is between 4 and 9 times that of other models.

### Improved neural turing machine analysis discussion

Figures 1D–F show the comparison of the training time of different models. four network structures are involved in this experiment as follows: (1) ordinary NTM. (2) NTM using Hard sigmoid activation function, which is referred to as H-NTM in the experiment. (3) NTM trained using adaptive curriculum learning strategy based on adaptive curriculum scaling, referred to as A-NTM. (4) H-NTM trained using adaptive curriculum-based scaling of curriculum learning strategies, referred to as AH-NTM. In Figures 1D–F, from top to bottom, are replication, addition, and multiplication, respectively. The models include AH-NTM, A-NTM, H-NTM, and NTM. Time units are minutes. Orange represents AH-NTM, yellow represents A-NTM, cyan represents NTM, and purple represents H-NTM. As can be seen from the figure, the time required to train NTM and A-NTM is very close, and the time required to train H-NTM and AH-NTM is very close. In the replication task, NTM takes 101 min; A-NTM takes 103 min; H-NTM takes 93 min; and AH-NTM takes 95 min. In the addition task, NTM takes 658 min; A-NTM both take 657 min; H-NTM takes 604 min and AH-NTM takes 600 min. In the multiplication task, NTM requires 1,829 min; both A-NTM require 1,801 min; H-NTM requires 1,688 min, and AH-NTM requires 1,694 min.

Figures 1D–F shows that the training time of H-NTM and AH-NTM is less than that of NTM and A-NTM takes more training time than AH-NTM. The specific figures are as follows: in the copying task, the training time of H-NTM is 8.0% less than that of NTM; the training time of AH-NTM is 6.0% less than that of NTM. In the addition task, the training time of H-NTM was reduced by 8.2% compared to NTM, and the training time of AH-NTM was reduced by 8.8% compared to NTM. In the multiplication task, the training time of H-NTM was reduced by 7.7% compared to NTM, and the training time of AH-NTM was reduced by 7.3% compared to NTM. In summary, the training time of the model is reduced by using the Hard sigmoid activation function instead of the sigmoid activation function in the NTM. The combination of the Hard sigmoid activation function and the adaptive course scaling-based course learning strategy reduces the training time of the model.

### Analysis and discussion of tensile properties of metal materials

**Figure 2:**
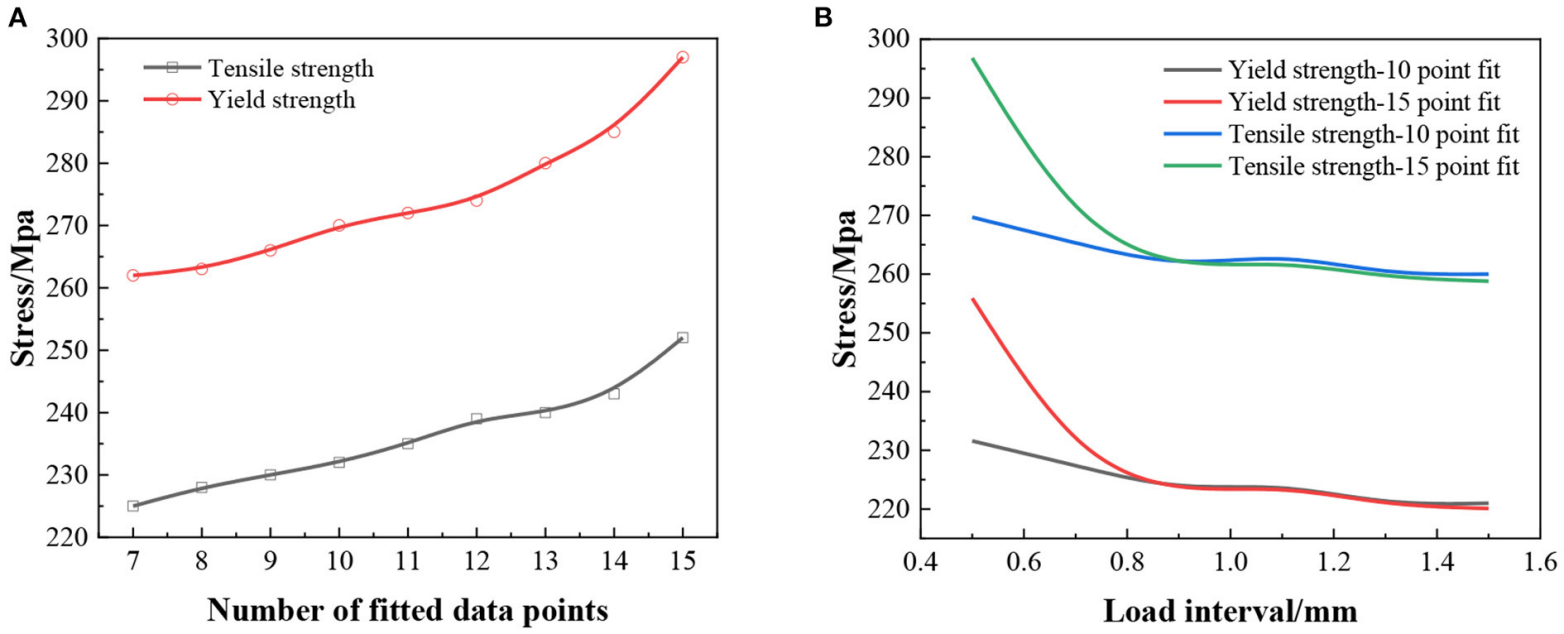
Calculated results of metal tensile properties, **(A)** material tensile properties for different data ranges at 0.5 mm press-in interval; **(B)** re-choice of fitted data.

In this experiment, the indentation interval is small and taken as 0.5, 0.55, 0.6, and 0.65 mm variables for the experimental study. The results of the second indentation test in this study were mainly influenced by the raised material surface. Its initial indentation load value is much larger than the true value, and the calculated distribution pattern of the characterized stress-strain data points deviates from the power-law intrinsic structure relationship of the aluminum alloy material. In order to improve the accuracy of the fitting results of this experiment. In this study, the results corresponding to the small initial indentation depth can be excluded. On this basis, the data points in the latter part of the study were selected to be more reasonably distributed for fitting. The smaller number of test points means that more initial data points are eliminated. For example, the 14 points represent the remaining 14 data sets after removing the stress-strain points obtained at the indentation depth of 10 μm. The indentation results at 0.5 mm indentation interval were used for the experimental analysis. The influence of the data selection range on the fitting and calculation results was investigated. The experimental study found that the most significant effect was caused by the first indentation at this working condition. As shown in Figure 2A, the stress of tensile strength and yield strength showed an increasing trend with the increase of fitting data points. As the number of points increases, the further away from the true value of the test. The true value of tensile test yield strength stress is 219.9 Mpa. The true value of the tensile strength stress of another group of tensile tests is 258.8 Mpa. This indicates that the indentation results with a small indentation depth are the main factor affecting the final calculation results. However, the number of test fitting points should be >10.

The yield strength obtained from this experiment using a 10-point fit was 4.32%. The relative error of the tensile strength was 4.17%. The relative errors of the initial results in the experiment were 15.17 and 14.68%. The comparison of the initial results with the 10-point fit indicates that reducing the choice of fitted data points in this case can significantly improve the accuracy of the calculated results. The 10-point fits were performed separately for the indentation results at different indentation intervals in the experiments. The variations of the calculated yield strength and tensile strength results are shown in Figure 2B. From the results, it can be obtained that the error of the initial indentation data is larger when the indentation interval is 0.5–0.7 mm. The error of the material tensile property results obtained after removing this section of data is significantly reduced and is closer to the true value. The data obtained were closer to the true value of the tensile test yield strength of 219.9 Mpa. another set of data was closer to the tensile test tensile strength of 258.8 Mpa. when the indentation interval was 0.8–1.5 mm, the accuracy of fitting the results using the default range was higher. The test stress data is infinitely closer to the true value with less error. Reducing the fitted data points will increase the calculated value of indentation and reduce its accuracy to some extent.

## Conclusion

Nowadays, metal material properties have become a hot research problem. Based on the assistance of neural Turing machine model, an improved neural Turing machine model is proposed in this paper. The model is applied to the exploration of tensile properties testing techniques for metallic materials. The model allows us to get the required results faster and more explicitly. It is found that the accuracy of fitting the results using the default range is higher when the press-in interval is 0.8 mm-1.5 mm. The specific findings of this study are as follows.

(1) In the experiments of neural Turing machine, the training time of four different models, RNN, LSTM, GRU and NTM, was compared for three different experimental tasks of replication, addition and multiplication. The analysis reveals that the training time of NTM is longer. In the replication task, the time required to train NTM is 2–5 times longer than the other models, respectively. In the addition task, it took 3–6 times as long to train as the others. In the multiplication task, it took 4–9 times longer to train than the others.
(2) In the experiments of the improved neural Turing machine, the training time was compared for four different models, NTM, H-NTM, A-NTM, and AH-NTM, for three different experimental tasks of replication, addition, and multiplication. The analysis shows the training time of H-NTM and AH-NTM is less than that of NTM. The training time of A-NTM is more than that of AH-NTM. The improvement of them reduces the training time of the models. In replication, the training time of AH-NTM is reduced by 6.0% compared to NTM, respectively. In addition, its training time was reduced by 8.8%, and in multiplication, its training time was reduced by 7.3%.
(3) When the indentation interval is 0.5–0.7 mm, the error of the initial indentation data is larger. This value is closer to the real value of the tensile test yield strength 219.9 Mpa and the real value of tensile test tensile strength 258.8 Mpa. When the indentation interval is 0.8–1.5 mm, the accuracy of fitting the results with the default range is higher, whose values are infinitely close to the true values. Reducing the number of fitted data points will increase the calculated value of indentation and reduce its accuracy to some extent.

## Data availability statement

The raw data supporting the conclusions of this article will be made available by the authors, without undue reservation.

## Author contributions

XC and WF validated their proposed ideas by designing experiments and analyzing the results in detail and then completed the paper writing. All authors read and approved the final draft.

## Conflict of interest

Author WF was employed by Huajin New Materials Research Institute (Guangzhou) Co., Ltd. and Guangdong Hongbang Metal Aluminum Co., Ltd. The remaining author declares that the research was conducted in the absence of any commercial or financial relationships that could be construed as a potential conflict of interest.

## Publisher's note

All claims expressed in this article are solely those of the authors and do not necessarily represent those of their affiliated organizations, or those of the publisher, the editors and the reviewers. Any product that may be evaluated in this article, or claim that may be made by its manufacturer, is not guaranteed or endorsed by the publisher.
